# Editorial: AI and robotics for increasing disaster resilience in modern societies

**DOI:** 10.3389/frobt.2026.1847221

**Published:** 2026-05-15

**Authors:** Jane Jean Kiam, Anastasios Dimou, Ron Alford, Armin Wedler, Yuichi Ambe

**Affiliations:** 1 Institute of Flight Systems, Aerospace Engineering, University of the Bundeswehr, Munich, Germany; 2 Centre for Research and Technology Hellas (CERTH), Thessaloniki, Greece; 3 The MITRE Corporation, McLean, VA, United States; 4 Institute of Robotics and Mechatronics, German Aerospace Center, Munich, Germany; 5 Graduate School of Advanced Science and Engineering, Hiroshima University, Hiroshima, Japan

**Keywords:** articial intelligence, disaster resilience (DR), rescue, robotic, unmanned systems

## 1 Motivation for the research topic

Hazards are expected to occur with increasing frequency, severity, or both (European Commission, 2023). Climate change is amplifying the occurrence of extreme events such as floods and wildfires. For instance, severe river floods are projected to more than triple in some regions by the end of the century (Alfieri et al., 2015), while rising thermal anomalies are expanding wildfire-prone areas (Jacome Felix Oom et al., 2022). At the same time, the increasing exposure of urban areas and wildland-urban interfaces is significantly elevating risks to human life, critical infrastructure, and emergency accessibility (Jacome Felix Oom et al., 2022; Dottori et al., 2023; van Ginkel et al., 2022). In addition, non-climate-related disasters, such as earthquakes, volcanic eruptions, and industrial accidents, pose growing threats due to rising population density in vulnerable regions (Guan et al., 2022; Merz et al., 2020). These developments underline the urgent need to strengthen disaster resilience, not only in terms of anticipation and prevention, but also in response to unavoidable events. In such scenarios, rapid and effective disaster response is critical to minimizing loss of life and limiting damage to essential infrastructure.

### 1.1 Unmanned systems for disaster resilience

Several previous publications have highlighted how unmanned systems can be used for disaster management. Erdelj and Natalizio (2016) point out clearly the three stages in disaster management where Unmanned Aerial Vehicles (UAVs) can be of essential support in: 1) early warning through sensor-based remote sensing, 2) disaster assessment with real-time monitoring of the disaster area, and 3) disaster response by being the communication nodes in a Wireless Sensor Network (WSN) or by transporting first aid to impervious areas. Guibaud et al. (2024) report on a real-world deployment of a remote-controlled ground robot for risk assessment during fire at Notre Dame Cathedral. A more recent scoping review by Mohd Daud et al. (2022) focuses on field reports of real-world deployments of UAVs, analyzing studies and experiments conducted using real-world data. In most scenarios, UAVs were used for disaster assessment by delivering images or by mapping the disaster area, including also three-dimensional mapping. Damage assessment in impervious areas was identified as the main advantage of UAVs in disaster management. Besides, UAVs can locate missing person(s) in Search and Rescue (SAR) operations faster, especially in snow-covered terrains.

### 1.2 AI for disaster resilience

Alongside the increasing risks associated with disasters, advances in artificial intelligence (AI) are creating new opportunities to enhance disaster resilience. Beyond enabling robots to act as sensor payload carriers for situational awareness (Verykokou et al., 2018), the integration of AI facilitates increased autonomy in decision-making. For example, prior work has demonstrated automated planning for complex rescue missions using unmanned autonomous platforms (Patra et al., 2019; Bit-Monnot et al., 2018). Such capabilities enable more effective operations, thereby improving operational efficiency without requiring additional human involvement in the decision-making loop.

Recent advancements in Large Language Models (LLMs) have also enable automated context extractions for disaster management (Xu et al., 2025; Chen et al., 2026), but also for seamless disaster response, in which operators command unmanned assets in natural language through LLM-powered interfaces (Döschl and Kiam, 2025).

## 2 Objectives of the special session

While robotics and AI were often Research Topic of scientific publications, targeted applications often vary. Often, the application in disaster relief plays only a marginal role, for example, being one of many example use cases for performance benchmarking. This special session addresses exactly recent advancements in robotics and AI in view of their use for increasing disaster resilience. Research Topic of interest include.

1. Novel sensor techniques and sensor fusion algorithms to be integrated on unmanned vehicles deployed for disaster response;
2. AI algorithms, frameworks, and systems for automated planning, sequential decision-making, multi-agent coordination etc. of unmanned vehicles in disaster areas;
3. Algorithms and methods for motion control of robots to be deployed in disaster areas (to overcome the physically challenging environment at a disaster site, e.g., uneven grounds due to debris for ground vehicles, stormy weather for aerial vehicles, etc.);
4. Collaborative capabilities for improved interaction of humans and unmanned vehicles in shared spaces;
5. Reporting on field validation tests for unmanned technologies in realistic environments and review articles.

### 2.1 Submissions

Accepted publications cover a wide spectrum of Research Topic. Yamauchi et al. focus on the design and development of an innovative robot demonstrator, namely, the 3.6 m long Dragon Firefighter (DFF), capable of extinguishing fire with onboard nozzles. The DFF has achieved stable manual flight, at the time the publication was submitted. In contrast, Tamura and Kamegawa addresses the control of snake robots on soft surfaces, which are highly relevant in disaster scenarios where terrain surface can vary significantly with respect to hardness. The developed control loop considers tactile feedback from different surface conditions, and uses a Central Pattern Generator (CPG) network to optimize coordination of the joints during locomotion.

Focusing on a more conventional UGV platform, Zafar et al. extend beyond isolated control loop by integrating hand-gesture-based tele-operation and YOLO-based victim detection for more intuitive human-robot interaction in the operational pipeline for search-and-rescue missions. Additionally, Moosavi et al.; Döschl et al. address multi-robot operations. Moosavi et al. investigate path planning for multiple snake robots in rescue scenarios and demonstrates a functional coordination in a simulation environment. Meanwhile, Döschl et al. focus on symbolic planning for multiple aerial robots, providing validation in photorealistic simulation environments and outlining a pathway toward integration with real robotic hardware.

### 2.2 Outlook

This special session brings together a Research Topic of recent state-of-the-art research contributions aimed at enhancing disaster resilience through the integration of AI and unmanned platforms. With the rapid advancement of AI, particularly in LLM-driven applications enabling increasingly sophisticated reasoning and decision-making capabilities (Webb et al., 2025), as well as recent progress in humanoid robotics (Han et al., 2025), the field is entering a phase of accelerated innovation. These developments are expected to continuously unlock new opportunities and research breakthroughs in the application of intelligent systems for disaster resilience.
